# Modeling tree diversity, stand structure and productivity of northern temperate coniferous forests of Mexico

**DOI:** 10.7717/peerj.7051

**Published:** 2019-08-14

**Authors:** José Návar

**Affiliations:** Division de Estudios de Posgrado e Investigación, Tecnologico Nacional de Mexico/Instituto Tecnologico de Ciudad Victoria, Ciudad Victoria, Tamaulipas, Mexico

**Keywords:** Weibull distribution, Diversity indices, Parameters of the three-dimensional canopy structure, Abundance models, Growth and yield model, Stand scale, Thinning, Above and below biomass productivity, Mixed coniferous forests

## Abstract

There is increasing evidence complex forest structure and tree diversity correlates positively with the productivity of forest ecosystems. However, there is little quantitative information regarding the effect of these factors on stand productivity of northern temperate coniferous forests of Mexico. This study aimed to test the hypothesis tree diversity and canopy structure positively associates with forest productivity. Parameterization of tree diversity, stand structure and productivity were carried out on dasometric data from 36 permanent sampling plots re-measured in 1982, 1993, and 2004. Statistical analysis of stand parameters tested the null hypothesis. Statistical relationships revealed well-balanced canopy strata and imbalanced diameter structures positively correlated with stand productivity. Tree diversity was also positively linked with stand productivity, but the effect appeared to be most important in the early to intermediate stages of succession. Further research is required to understand the long-term effects of tree diversity and canopy structure on stand productivity. These preliminary observations stress the importance of prescribing silvicultural practices that maintain the three-dimensional structure of stands and diversity of forest canopies that aim to preserve ecosystem function, diversity, and productivity.

## Introduction

Native forests with high tree species diversity are more productive than less diverse forest communities (Tilman et al., 1997). This finding is also consistent across other plant communities; e.g., for American and European grasslands (Tilman et al., 1997; Hector et al., 1999). Along with tree species diversity, structural complexity (e.g., the variability of the three-dimensional spatial assemblage of trees and other structural elements within forests) also correlates positively with forest productivity (Ishii et al., 2000; Ishii, Ford & Dinnie, 2002). Phenological complementarity, the asynchrony (differential timing) in the use of resources and the differential growth patterns of species may explain how diversity influences ecosystem function (Stevens & Carson, 1999; Stevens & Carson, 1999a). Structural complexity increases forest productivity by optimizing complementary resource utilization among plant species as well (Hooper & Vitousek, 1997). Native forests maintain high tree species diversity usually associated with structurally complex stands. In contrast, very young or old forest stands and plantations are featured by low tree diversity and structurally simple forests. Changes of habitat structure during stand development partially explain these forest features (Carey & Wilson, 2001).

Several forest management methods aim to preserve low tree diversity and structurally simple tree populations simulating forest plantations. A consistent decline in forest productivity and the impaired ability to buffer against perturbation are some of the consequences of these silvicultural prescriptions on native forest communities (Schulze & Mooney, 1994). Individual trees and forest populations may be particularly damaged by reduced diversity and limited stand structure complexity by climatic events with a low probability of occurrence such as frosts, acute dry spells associated with unusual heat waves, cold windstorms, pests and diseases, acid rain episodes, shifts in soil fertility, among others. For example, intense drought episodes associated with sporadic heat waves of different time scales are contemporary perturbations impacting negatively forest structure and productivity negatively around the globe (Bonan, 2008; Raffa et al., 2008). High temperatures associated with soil dryness beyond normal thresholds are responsible for the pervasive tree mortality rates over the western North American forests, the tree mortality pulses by bark beetle population eruptions, and the increased wildfire activity elsewhere ( Van Mantgem et al., 2009; Allen et al., 2010; Návar, 2008). Based on these observations, silvicultural prescriptions of native forests must aim to increase tree diversity and structural complexity in order to maintain the ability to buffer against these kinds of modern perturbations induced by increasing climate variability and/or climate change (Hunter, 1999; Bonan, 2008). In the meantime novel silvicultural approaches are developed in a likely future with accelerated changes (O’Hara, 2016).

In spite of this scientific evidence, research has yet to be conducted on northern temperate coniferous forests of Mexico to better understand the effect of tree diversity and structural complexity on stand productivity. Such insights are necessary for the development of effective silvicultural guidelines. This research tested the null hypothesis that tree diversity and structural complexity is not correlated with stand productivity. Then, the objectives of this research were: (a) to parameterize tree diversity, stand productivity and structural complexity and (b) to statistically associate stand productivity, tree diversity, and structural complexity with the goal of prescribing coherent forest management practices as they may control stand productivity and resilience to contemporary perturbations in the northern coniferous forests of Mexico.

## Materials and Methods

### Study area

This research was conducted in the main core of the Central Portion of the Western Sierra Madre Mountain Range of Durango, Mexico. The region is characterized by a cold-temperate climate, with annual average rainfall and temperature of approximately 1,000 mm and 14 °C, respectively. Soils are classified as Litosols, Rendzins, Leptosols, and Regosols. Soils are shallow (<30 cm in depth), rich in acidic bases, and high in organic matter content. Unevenly aged, mixed coniferous forests with *Pinus cooperi*, *Pinus durangensis*, *Pinus leiophylla*, *Pinus teocote*, *Quercus sideroxylla*, *Arbutus xalepensis*, *Arbutus chiapensis*, *Juniperus spp*, *Alnus accuminata*, and other less frequent tree species distribute along this mountain range. Permanent sampling plots are located at an altitude of 2,450 m above sea level.

### Permanent plots and silvicultural treatment

In this forest range, 36 permanent sampling plots were demarcated in 1967 in the municipality of San Dimas, Durango, Mexico. Each permanent plot was 100 m × 100 m quadrat with a measurement sub-quadrat of 50 m × 50 m located at the center for continuous inventory of dasometric parameters. Plot selection was conducted following a complete randomized design with six treatments and six replicates. Silvicultural treatments consisted on removing basal area at different intensities; (0) 0% or control, (1) 20%, (2) 40%, (3) 60%, (4) 80%, and (5) 100% or clear-cutting. Dasometric data recorded during the forest inventories of 1982, 1993, and 2004 were used to derive parameters of stand productivity, tree diversity, and stand structural complexity.

### Forest productivity

Forest productivity was estimated for below ground biomass (coarse roots, BGB) and aboveground (stems, branches, and leaves) standing biomass (AGB), hereon denoted productivity, for three periods (1982–1993, 1993–2004, and 1982–2004) using allometric equations developed by Návar (2009) for large pine trees (*AGBp* = 0.0726*D*^2.4459^) and for oak trees (*AGBq* = 0.0768*D*^2.4416^). Allometric equations estimated standing AGB for all coniferous and all broadleaf tree species. Assessments were compared to independent calculations of biomass by the classical physics equation M = VP; where M = biomass; V = volume, and P = wood specific gravity, wsg. The Schumacher volume equation derived previously by Contreras-Aviña & Návar-Cháidez (2002) was used to evaluate timber volume. According to a literature review, the average wsg for pines was 0.43, and for oaks was 0.60 g cm^−3^ (Davalos, Wangaard & Echenique-Manriquez, 1977; Compean-Guzman, 1996). This comparison tested the consistency of both AGB estimates . The Clark et al. (2001) technique was used to estimate stand productivity following method 2. Using the allometric equations, total AGB was estimated for 1982, 1993, and 2004. Forest productivity was derived from (AGB1993-AGB1982)/11 for the first period; (AGB2004-AGB1993)/11 for the second; and (AGB2004-AGB1982)/22 for the full period of observations. The same procedure was employed to evaluate coarse root biomass productivity, but using the coarse root equation (BGB = 0.016D^2.668^) reported by Návar (2009).

### Tree diversity

Tree diversity was parameterized by fitting nine diversity indices for data recorded during each of the three forest inventories for each quadrat: three diversity indices based on species richness, one based on abundance, and four based on the proportional abundance of species. The diversity indices employed were species richness (S), Margaleff (Mg), Menhinick (Mn), Shannon Weiner (SW), Brillouin (Br), Simpson (Si), McIntosh (Mc), and Berger–Parker (B-P).

Four models fitted the abundance-diversity relationship for each plot for each of the three forest inventories to explain partition of limiting resources within the species community, allowing the drawing of conclusions regarding the successional processes that shape the productivity-diversity relationship. The equations describing the geometric series, the log series, the truncated lognormal distribution, and the broken stick model reported in McArthur (1957) and Magurran (2004) were fitted for each quadrat for each of the three forest inventories.

### Stand structure

Various measures of stand structure have been proposed for guiding forest management (Staudhammer & Lemay, 2001). In this research, the three-parameter Weibull distribution for the variables top height, H, and diameter at breast height, D, depicted well the tree-size distribution that portrays the three-dimensional structure of forests (Haan, 2003; Hahn & Shapiro, 1967). The conventional procedure of moments in software reported by Návar & Contreras (2000) evaluated the shape, *α*, scale, *β*, and location, ε, parameters of the Weibull density function for D and H for each forest plot for each of the three forest inventories.

### Stand attributes, diversity, and productivity

Linear and quadratic second-degree polynomial regressions fitted AGB productivity, P, to predict diversity, S, [(e.g.,  *S* = *a* + *b*(*P*) + *c*(*P*)^2^] for each of the two biomass components as well as for each of the three time periods using data at the quadrat scale. Comparisons of the coefficients of determination, *r*^2^, and the significance of the model determined the type of regression that best describes the tendency of these relationships. These statistics test the appropriateness of the Unimodal, U-shaped and monotonic models (Mittelbach et al., 2001). In addition, I tested whether the quadratic relationship reached a maximum or minimum within the observed range of forest productivity values. This test examines the null hypothesis that *c* = 0, and checks whether the data supports an intermediate maximum or a minimum number of species within the range of observed productivity. This test also provides information on whether the quadratic is a better fit than the monotonic increasing or decreasing diversity with productivity (Leibold, 1999). Quadratic relationships that showed a maximum, negative c coefficient were classified as hump-shaped, while those that showed a minimum, negative b and positive c coefficients, were classified as U-shaped. Relationships without a statistical significant maximum (hump-shaped) or a minimum (U-shaped) were classified as linear models since transformations can linearize any monotonic relationship. All nine diversity indices were regressed against below and aboveground standing productivity. Therefore, a total of 54 regressions were tested for linearity or non-linearity (9 diversity indices and 2 productivity component) for all 36 quadrats for each of the three time periods between forest inventories.

### The growth and yield model

Pines (*P. cooperi*) and oaks (*Q. sideroxylla*) are dominant tree species in these forest plots. Two types of timber growth and yield models were used to test the binary (pines and oaks) effect of tree diversity on forest productivity (Clutter et al., 1983; Vanclay, 1994). The first type of growth and yield model was constructed with the typical variables site index, basal area, the average age of pine trees, and the diversity component given by the density of oak trees/total stand density. The effect of the diversity component on timber growth and yield was tested by establishing the null hypothesis that the coefficient = 0. The second growth and yield model was developed individually for the six stands treated with clear-cutting (stands with no oak trees) and the rest of the forest stands (stands with oak trees of differential density). Timber volume projections over time by both types of models tested the null hypothesis that timber growth and yield are equal over time between mixed stands and mono-specific stands with only pine trees.

### Stand structure complexity and productivity

The null hypothesis of the effect of canopy structure on forest productivity was tested by fitting regression equations between the Weibull parameters (*α*, *β*, and ε) of the H and D stand parameters and forest productivity for both time periods. The probability of the regression coefficients tested e.g., Ho:B1 = 0 or Ha:B1 ≠ 0. If Ho is rejected, then structural features represented by the Weibull parameters of the canopy explain part of the variation in forest productivity. In addition, statistical relationships between H and D and forest productivity were developed.

## Results

### Forest stand attributes

Average stand timber basal area, volume, density, and root and aboveground biomass productivity components increased over time, unlike D and H, which oscillated erratically between time periods. Tree mortality, in-growth or recruitment, and likely errors in measurements of D and H accounted for part of the erratic averages of D and H. Pine trees dominated forest canopy in all 36 measured forest stands as basal area, stand density, timber volume, root biomass and aboveground standing biomass recorded larger statistics in pine, as compared to oak trees (Table 1).

**Table 1:** Average and confidence intervals (*p* = .05) for several dasometric parameters for each period of measurements of 36 permanent sampling plots established in Durango, Mexico.

	Oak Trees							Pine Trees						
Para meter	D	H	BA	V	Den	AGB	BGB	D	H	BA	V	Den	AGB	BGB
	(cm)	(m)	(m^2^ ha^−1^)	(m^3^ ha^−1^)	(No ha^−1^)	(Mg ha^−1^)	(Mg ha^−1^)	(cm)	(m)	(m^2^ ha^−1^)	(m^3^ ha^−1^)	(No ha^−1^)	(Mg ha^−1^)	(Mg ha^−1^)
	1982													
Mean	14.7	7.6	9.8	106.5	332	57.7	6.91	16.2	10.5	15.0	182.9	627	72.1	16.9
C.I.	1.6	0.9	2.4	27.3	85	16.6	0.005	1.3	0.9	1.2	19.3	150	6.8	0.005
	1993													
Mean	12.1	6.7	10.7	120.9	393	67.1	4.87	16.1	10.9	19.5	248.5	783	96.6	20.8
C.I.	2.0	1.1	2.7	32.0	104	19.2	0.01	1.0	0.8	1.9	21.7	176	8.0	0.003
	2004													
Mean	11.3	6.9	11.2	125.5	417	73.4	4.31	15.4	10.9	22.8	313.9	866	118.4	20.4
C.I.	2.0	1.2	3.0	34.7	111	21.0	0.01	0.9	0.7	2.0	26.5	169	9.4	0.002

**Notes.**

D diameter at breast height H top height BA basal area Den density AGB aboveground biomass estimates (AGB = aD^b^) RB root biomass estimates (RB = aD^b^) C.I. confidence interval

Allometric and physical equations provided compatible AGB biomass assessments for oak and pine trees. Therefore, the conventional procedure of biomass estimation using allometric equations for individual trees was used to evaluate stand productivity. Total AGB productivity had an average (± confidence interval) of 3.09 ± 0.72 Mg ha^−1^, 2.55 ± 0.77 Mg ha^−1^, and 2.82 ± 0.60 Mg ha^−1^ for the periods of 1982–1993, 1993–2004, and 1982–2004, respectively. Coarse root biomass productivity had an average (± confidence interval) of 0.17 ± 0.001 Mg ha^−1^; 0.01 ± 0.0001 Mg ha^−1^; and 0.08 ± 0.001 Mg ha^−1^, for the time intervals of 1993–1982, 2004–1993, and 2004–1982, respectively. The drought spell of the 1990s (1989–2001) recorded and reported by Návar (2015) may have reduced total AGB productivity from 3.09 to 2.55 Mg ha^−1^ y^−1^, as well as coarse root biomass productivity from 0.17 to 0.08 Mg ha^−1^y^−1^, between the time periods of 1993–1982 and 2004–1993, respectively.

Removal of the basal area played a significant role by enhancing below and aboveground productivity (*p* ≤ 0.05; Table 2). There was an increasing tendency towards below and aboveground biomass productivity with basal area removal, with coefficients of determination (*r*^2^) of 0.36, 0.04, and 0.23; and 0.48, 0.25, and 0.44 for 1982–1993, 1993–2004, and 1982–2004, respectively. According to the tendency of these relationships, BGB and AGB productivity was least in control treatments, accelerated in a non-linear fashion with basal area removal, reaching the highest values in stands treated with 100% basal area removal. Growth of juvenile forests and most importantly the recruitment of young trees in forest openings partially explained the augment of productivity with increased basal area removal.

**Table 2:** Mean and confidence intervals of total below and aboveground biomass productivity with basal area removal for 36 permanent sampling plots in Durango, Mexico.

Basal area removal (%)	1993–1982		2004–1993		2004–1982	
	AGB	BGB	AGB	BGB	AGB	BGB
0	2.69 ± 0.52	0.82 ± 0.18	2.49 ± 1.10	0.82 ± 0.37	2.59 ± 0.81	0.84 ± 0.28
20	2.37 ± 0.40	0.78 ± 0.14	3.31 ± 1.34	1.05 ± 0.44	2.84 ± 0.47	0.93 ± 0.17
30	1.86 ± 0.75	0.63 ± 0.26	1.07 ± 0.24	0.40 ± 0.09	1.46 ± 0.25	0.58 ± 0.09
50	4.36 ± 1.15	1.30 ± 0.38	2.69 ± 0.51	0.85 ± 0.18	3.53 ± 0.83	1.13 ± 0.28
70	2.56 ± 0.03	0.83 ± 0.02	2.43 ± 0.45	0.78 ± 0.16	2.49 ± 0.24	0.82 ± 0.09
100	4.68 ± 1.06	1.39 ± 0.35	3.33 ± 0.51	1.08 ± 0.18	4.00 ± 0.79	1.23 ± 0.27
Mean	3.08 ± 0.52	0.96 ± 0.18	2.55 ± 0.61	0.83 ± 0.21	2.82 ± 0.57	0.92 ± 0.20
C.I.	2.47 ± 0.41	0.25 ± 0.11	2.04 ± 0.49	0.20 ± 0.17	2.26 ± 0.45	0.19 ± 0.16

### Tree diversity

*P. cooperi* regenerated in stands with 100% of the basal area removed, as well as several other stands with smaller openings. *Q. sideroxylla* also colonized canopy openings caused by tree mortality. Assemblages of seral tree species recorded during inventories were composed by *P. teocote*, *P. leiophylla*, *P. ayacahuite*, *Arbutus spp*, *Alnus spp*, *Juniperus spp*, *Pseudotsuga spp*, and *Q. sideroxylla*. Thus, oaks colonized forest openings and regenerated well under the canopy of pine trees in treated stands, as well. Tree density and species richness increased over time. Recruitment of individuals of secondary pine and oak species (*P. ayacahuite*, *P. leiophylla*, and *Q. sideroxylla*) enriched stand density and tree diversity. Most secondary species of succession are shade-tolerant and colonize well-stocked forest stands, as observed by mortality and canopy overlap between trees of the dominant pine species, *P. cooperi*. In control plots, *Pseudotsuga menziesii* colonized overstocked stands from 1993 to 2004. Species richness was accelerating during this period although the rate had decelerated since species richness, S, was increasing on the average from eight to 11 species per plot. Stand density was still increasing although the rate has decelerated notoriously causing the diversity indices to oscillate erratically over time (Table 3).

**Table 3:** Mean and confidence intervals of diversity indices estimated for 36 permanent sampling plots in Durango, Mexico.

Para-meter	Density	Diversity indices							
	(No ha^−1^)	S	Mg	Mn	SW	Br	Si	Mc	B-P
	1982								
Mean	1065	9.25	1.50	0.59	1.36	1.07	3.12	0.41	2.08
C.I.	138	0.73	0.13	0.05	0.15	0.12	0.47	0.05	0.27
	1993								
Mean	1305	9.47	1.48	0.54	1.34	1.09	3.06	0.40	2.03
C.I.	149	0.80	0.14	0.05	0.16	0.13	0.45	0.05	0.25
	2004								
Mean	1439	10.56	1.63	0.57	1.39	1.13	3.12	0.41	2.03
C.I.	139	0.89	0.15	0.05	0.15	0.13	0.43	0.05	0.23

**Notes.**

S species richness Mg Margalef Mn Menhinick SW Shannon & Weiner Br Brillouin Si Simpson Mc McIntosh B-P Berger-Parker C.I. confidence intervals (α = 0.05)

Stand density and species richness increased over time in all treated quadrats, except for density in stands with 100% basal area removal (Table 4). This tendency resulted from tree regeneration in gaps originated by basal area removal and the recruitment of shade-tolerant species under the canopy of remnant trees. In plots treated with 100% basal area removal, after reaching a maximum density of 1800 trees ha^−1^ with an average D of 18. one cm in 1982, intrinsic competition between individuals of *P. cooperi* caused considerably tree mortality, reducing density to 1300 trees ha^−1^ with an average D of 18. five cm in 1993. Shade-tolerant tree species *P. ayacahuite*, *Q. sideroxylla*, *P. teocote*, *Junniperus spp*, *Alnus spp*, *Arbutus spp*, and *P. leiophylla* colonized overstocked stands accelerating stand density to 1400 trees ha^−1^ during 2004 through the reduction of *P. cooperi* trees. Intra-specific competition of *P. cooperi* reduced stand density while recruitment of shade-tolerant species increased stand density, as well as tree diversity.

**Table 4:** Means and confidence intervals of diversity indices estimated for six silvicultural treatments conducted on permanent sampling plots in Durango, Mexico.

Basal area removal	Density	Diversity indices							
	(No ha^−1^)	S	Mg	Mn	SW	Br	Si	Mc	B-P
1982									
0	881	9.67	1.61	0.66	1.44	1.11	3.18	0.44	2.17
20	942	9.50	1.56	0.62	1.42	1.12	3.15	0.44	2.05
40	895	9.33	1.55	0.63	1.49	1.16	3.75	0.45	2.29
60	988	9.33	1.51	0.59	1.44	1.13	3.22	0.43	2.10
80	855	9.83	1.64	0.67	1.51	1.16	3.65	0.47	2.46
100	1826	7.83	1.12	0.37	0.87	0.73	1.78	0.24	1.37
1993									
0	1131	9.67	1.57	0.61	1.44	1.14	3.14	0.44	2.08
20	1356	9.33	1.47	0.55	1.44	1.16	3.18	0.44	2.02
40	1290	10.17	1.62	0.60	1.46	1.17	3.62	0.44	2.22
60	1433	9.67	1.51	0.55	1.42	1.15	3.29	0.43	2.22
80	1339	9.00	1.42	0.54	1.44	1.16	3.33	0.45	2.25
100	1279	9.00	1.28	0.40	0.87	0.73	1.80	0.24	1.37
2004									
0	1285	11.17	1.79	0.66	1.49	1.19	3.24	0.44	2.07
20	1464	10.33	1.62	0.58	1.48	1.21	3.25	0.45	2.03
40	1417	11.50	1.81	0.63	1.50	1.22	3.66	0.44	2.25
60	1569	10.17	1.56	0.54	1.44	1.19	3.36	0.43	2.19
80	1479	9.67	1.51	0.55	1.43	1.16	3.22	0.44	2.18
100	1419	10.50	1.51	0.46	0.99	0.83	2.01	0.27	1.45

At the plot scale, within a time interval, the diversity indices of species richness, Shannon & Weiner, Brillouin, Simpson, McIntosh, and Berger-Parker had a tendency to diminish over time. This was the result of increasing stand density and the decline of the rate of species richness with increasing basal area removal. In general, removing 100% of the basal area resulted in the smallest diversity indices (Table 4).

Most diversity indices however increased over time within each plot for all periods of recording data (Table 4). This tendency is noted primarily for all diversity indices in stands treated with the removal of 100% of the basal area. An increasing number of seral species colonized stands and the strong intra-specific competition of individuals that reduced the density of the pioneer species *P. cooperi* explained this tendency. Overcrowding processes of ecological competition reduce the density of dominant and increase the density of secondary species of succession. Although plots treated with total clearing increased species richness and revived stand density due to the regeneration of seral species, the diversity indices have not yet recovered when contrasted with those of other plots.

### Diversity-Abundance models

The effect of basal area removal on the diversity—abundance structure is demonstrated by the number of significant null hypotheses accepted (*p* ≥ 0.05) for the geometric, log series, the lognormal, and broken stick models. Although there is no overall tendency for all of the accepted null hypotheses, it is clear that plots treated with 100% basal area removal have not yet recovered the abundance-diversity structure, since these stands had only 25% of all potential accepted null hypotheses. The rest of the stands had at least 50% of the accepted null hypotheses of the four fitted diversity-abundance models.

Diversity-abundance of most quadrats is developing quite effectively since the geometric series (a model adapted well to pioneer stages of succession) has the least number of null hypotheses accepted. Fitting tests accepted at least 50% of the null hypotheses of the broken stick model and 25% of the log series and lognormal model; three diversity-abundance models describing well the advanced stages of succession. That is, the differential abundance of most tree species is being reduced over time in these forests.

### The canopy structure

The Weibull distribution fitted well D and H distributions for 92.5% of plots (probability of Kolmogorov-Smirnoff ≥ 0.05). Parameters describing the canopy structure showed the H structure developed over time from a nearly J inverse (*a* = 1.89 for 1982 and *a* = 2.19 for 2004) to a bell-shaped distribution with an increasing dispersion over time (*b* = 11.63 for 1982 and *b* = 14.44 for 2004). The smallest top height of trees was in the range of 2 to 3 m. Although Weibull parameters varied over time, the mean H (standard deviation) remained quite constant throughout the measurements. It was 9.01 m (2.61 m) for 1982, 8.88 m (2.56 m) for 1993, and 8.91 m (2.62 m) for 2004. Using the Weibull probability density function, there was a 74% and only a 13% probability of finding trees with 5 ≤ H ≤ 20 and H ≥ 20 m, respectively. *Quercus spp*, *Arbutus spp*, and *Alnus spp* occupied the lowest strata, H ≤ 10 m, followed by oaks in the middle strata with an average top height of 11.3 m (±4.3 m), and pine trees in the upper strata with an average top height of 13.0 m (±5.7 m). In forest openings, pioneer pine trees (*P. cooperi*) colonized well these places. Shade-tolerant pine trees (*P. ayacahuite* and *P. leiophylla*) established well under the canopy of other pioneer pine species.

Diameter structures also developed towards a bell-shaped, symmetric distribution but the rate of change was quite small (note the shape parameters of the Weibull distribution). Diameter structure contracted over time, in contrast to top height structures. Using the Weibull density function, there was a 40% and only a 28.7% probability of finding trees with 10 ≤ D ≤ 20 cm and D >20 cm, respectively. The probability of finding trees with D >30, 40, and 50 cm reduces to 10, 2, and 1%, respectively. Oaks recorded an average diameter and basal area of 17.0 cm (11. five cm) and 11.9 ± 7.7 m^2^ ha^−1^ while pine trees, characterized by *P. cooperi*, had an average diameter of 17.7 ± 10. 1 cm and basal area of 16.1 ± 7.7 m^2^ ha^−1^.

Trees in plots treated with 100% basal area removal reached a bell-shaped distribution for H and D in the last inventory. The variance of H and D was the smallest in this treatment, mimicking a well-stocked forest plantation. In contrast, untreated forest stands developed towards a J-inverse diameter distribution and to a bell-shaped H distribution. Mortality of large trees, recruitment of pioneer tree species in openings, recruitment of shade-tolerant tree species below canopies of large trees, and other unaccounted factors such as random tree mortality by competition enhanced canopy structure complexity. This tendency was also consistent for stands with 20% and 40% basal area removal.

### The relationship between tree diversity and forest productivity

Most relationships between diversity indices and productivity did not present statistical significance (*p* > 0.05), with the exception of the monotonic increment of the Shannon Weiner and Brillouin indices with increasing productivity (*p* ≤ 0.0024). Correlations were positive but following a logarithmic tendency, with small coefficients of determination, *r*^2^ < 0.10. All diversity indices increased, as productivity did, but the variation was so large that resulted in a lack of statistical significance. This was probably due to the fact that between 7 and 12 tree species colonized these forests with only two species dominating stand density (*Q. sideroxylla* and *P. cooperi*). Only a few individuals of other less common tree species (*Alnus spp*, *Arbutus spp*, and *Pseudotsuga spp*) were inventoried in few quadrats.

### Timber growth and yield model

**Figure 1:**
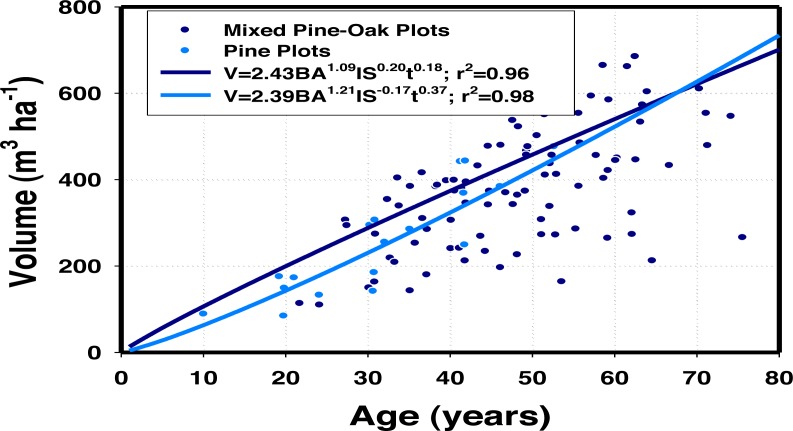
A simple two-dimensional representation of volume growth for pine-oak and pine stands in 36 permanent sampling plots in Durango, Mexico.

The relative density of oaks increased significantly timber growth (*p* ≤ 0.05) highlighting the relative importance of tree diversity on stand productivity (Table 5). Timber volume (m^3^ ha^−1^) was a function of basal area (BA), site index (SI), the average age of pine trees (t), and oak density/stand density (IQ). However, IQ was better correlated to timber growth than the conventional SI derived from the Schumacher model fitted to the relationship of the top height of dominant pine trees and age. A simple representation of the two timber growth models, one for only pine forests and the second for mixed pine-oak stands is depicted in Fig. 1. Timber growth reached higher yields in mixed stands in contrast to mono-specific pine stands, although the effect appeared to be temporally limited. For the range of observations, timber growth was larger in mixed stands with the maximum deviance of 58 m^3^ ha^−1^, reached at 25 years of stand projection. After 65 years of stand development, timber growth appeared to be higher in mono-specific pine stands.

### Canopy structure and stand productivity

In highly productive plots, the height of most trees was restricted 5 ≤ H ≤ 15 m, and the horizontal distribution of trees appeared to depict well a J–inverse shaped distribution. In the least productive plots, on the contrary, the H distribution increased its variation (2 ≤ H ≤ 25 m) and was less balanced; the diameter distribution returned to a right-skewed distribution, indicating that the H distribution of trees appeared to be more important in determining AGB productivity.

## Discussion

Tree species must use niche differentiation as a deterministic rather than a random process in order to increase forest productivity (Ishii, Ford & Dinnie, 2002). It is also termed complementary effects that enhance the performance of forest communities (Tilman et al., 1997). Mixed forest communities make use of resources (light, soil, and water) available in the different strata optimizing the use of available resources, as was the case for these northern temperate forests of Mexico. Variability of vertical and horizontal structures also control stand productivity as it has been found for other forests (Ishii, Ford & Dinnie, 2002). This factor has been related to tree diversity as well. The canopy structure determines the distribution of light in forest communities and therefore controls forest productivity (Parker, 1995). Consistent findings with these statements are noted for northern forests of Mexico in this research as tree diversity and the structural complexity of forest canopies are positively correlated well with AGB productivity. However, the relationships and the mechanisms that shape these interactions are quite complicated to describe and require further research. These northern temperate forests contain between nine and 12 tree species but with only two dominant forest species; they are characterized by having skewed diameter distributions and tree density consistently increasing over time. Aboveground biomass yield (e.g., the integration of productivity, Mg ha^−1^ y^−1^) is higher in native forests when oaks make up 20% of the total stand density (e.g., at 50 years of the rotation cycle, pine forests may contain a mean of 420 Mg ha^−1^ while mixed pine-oak forests would have 460 Mg ha^−1^). The difference of 40 Mg ha^−1^ represents about 10% of the mean aboveground biomass productivity of uni-specific forest plots at 50 years of age. Part of this productivity is in the oak trees themselves. Hiura (2001) observed that pine density was not significantly correlated with the growth of *Quercus crasipula* trees in temperate forests of Japan. Additional measurements are required to test the null hypothesis that H and D grow at a faster rate in mixed than in mono-specific forest stands.

**Table 5:** Timber growth and yield models considering the inclusion of oak density as explanatory variable for 36 permanent sampling plots of Durango, Mexico.

V=b0*BA^b1^*Si^b2^*t^b3^*IQ^b4^;*r*^2^ = 0.95			V=b0+b1(BA)+b2(SI)+b3(t)+b4(IQ);*r*^2^ = 0.96		
Para-meter	Estimate	Probability	Parameter	Estimate	Probability
b0	1.3782(0.21)	0.0001	b0	−83.14(18.59)	0.0001
b1	1.0342(0.03)	0.0001	b1	13.08(0.45)	0.0001
b2	0.0060(0.06)	0.9224	b2	−1.43(0.64)	0.0269
b3	0.2723(0.03)	0.0001	b3	1.88(0.24)	0.0001
b4	0.0290(0.006)	0.0001	b4	38.14(14.06)	0.0078

The binary effect of tree diversity (the ratio of oaks to pines) must be tailored during the harvesting rotation cycle with the promotion of silvicultural practices that aim to maintain tree diversity in equilibrium with abundance. The effect appears to be nonlinear in this short period of time studied. The nonlinear effect has a slope coefficient, B4 <1.0 stressing the limited effect of the ratio of oak to pine when controlling stand productivity. Oaks appear to grow at a slower pace than pines (Návar, 2014). Then, the index that optimizes forest growth must be further investigated with a secondary main null hypothesis that the ratio is independent of the tree dimensions. In these forests, I had never seen a fully stocked plot or stand with two strata: one fully stocked by oaks and pines, oaks dominating the lower and pines dominating the upper strata. Should there be forest stands like this one, the ratio would reach the highest figure of 50%. A comprehensive commercial forest inventory in the region, as well as the Mexican Forest Inventory for the state of Durango, shows forest stands with commercial trees, pines dominate abundance statistics with 75%, while oaks account for nearly 20% and the remaining tree density. Other tree species composing the forest are *Alnus spp*, *Junniperus spp*., *Pseudotsuga spp*., among others (Graciano, 2001; Návar, 2014). Then, the recommended silvicultural guideline would be to manage forest stands with a ratio of oaks of between 15 to 25% of the total tree density present in the forest community; as it has been noted for other native forests (Franklin et al., 1989; Kelty, 1992; Berg, Brown & Blessing, 1996). Kelty (1992) also contrasted the productivity of monocultures and mixed forests and strongly recommended the maintenance of tree diversity as they increase AGB productivity.

The number of tree species and the structural complexity had been recovered after harvesting 100% of the tree community reaching previous values. In addition to the pioneer *P. cooperi* tree species, recruitment of other 7 tree species took place between 1967 and 1982. Succession in this short period of time enriches tree diversity having an average number of tree species similar to the originally mixed stands, with less than 11 tree species per plot in 2004. These forests take only 40 years to recover the previous tree diversity S-species values. This short period of time is less than one harvesting intervention cycle in these forests. However, simple projections of the Shannon–Weiner diversity index or the broken stick diversity-abundance model over time indicates that tree diversity would return to compatible previous diversity index figures in about 140 years. That is, the equilibrium between stand diversity and species abundance, which can be contrasted with other mixed stands, takes longer than previously anticipated. Continuous tree harvesting at the same pace would eventually lead to unbalanced diversity-abundance of tree species, which eventually would lead to reduced productivity by simplifying tree diversity of native temperate forests. Then, the recommended silvicultural prescriptions would be to manage native pine forests with the typical harvesting cycle but allowing the tree diversity to remain balanced throughout the management cycle; by promoting fast in-growth of the harvested tree species. This could be accomplished by modifying the selection system of individual trees for a selection system based on forest stands. The size, shape, and location of the forest stand to be totally harvested by the new selection system are a matter of further research.

Conventional stand density guidelines could provide some insights into the management of these forests. Most pine species are shade intolerant and require full sunlight to carry photosynthesis efficiently. Tree growth drives intrinsic competition by demography leading to tree mortality by sunlight as the limiting resource in dominated trees (Clutter et al., 1983; Stevens & Carson, 1999). The self-thinning mechanism described by the −3/2 law evaluates the number of trees remaining as they grow in size (Huston, 1994) and the Reineke density index describes the optimal stand density (Clutter et al., 1983). These techniques work well for pioneer pine species but they fail to provide insights into the correct stocking for secondary species of succession. Therefore more research is recommended to understand better factors leading to the establishment of secondary tree species in these forests. That is, the self-thinning mechanism collapses when worked out at the scale of the native forest; in the presence of diverse and structural complex forests, as was the case for these native forests. In these forests, shade-tolerant tree species *Pseudotsuga menziensii*, *Pinus ayacahuite* and *Pinus teocote* are being recruited under the canopy of dominant *P. cooperi* pine species. To complicate the self-thinning mechanism at the native forest scale, tree recruitment by shade-tolerant and shade-intolerant oaks dominated by *Q. sideroxylla* also colonize and develop well under the canopy of 100% basal area removal pine stands dominated by *P. cooperi* trees as well as in forest clearings by the harvesting of the large pine trees of other plots. Therefore, there is a need for developing stand density guidelines for complex native coniferous forests.

The ability of *P. menziensii*, *P. ayacahuite*, and *Q. sideroxylla* to colonize and develop well under the canopy of *P. cooperi* may be related to deterministic rather than to stochastic processes of several local and temporal scales. Tree species must use niche differentiation as a deterministic process in order to increase stand productivity (Ishii, Ford & Dinnie, 2002; Tilman et al., 1997). Facilitation among tree species and vertical stratification may play important roles (Hartley, 2002). In this process, niche differentiation appears to determine species assemblages and community dynamics, as proposed before for tropical forests (Wright, 2001). Compartmentalization may help to explain niche differentiation between tree species when using e.g., soil resources. Roots of pine trees grow preferentially at upper soil compartments while roots of oaks exploit more commonly deeper soil profiles. These findings have also been reported for other oak trees of United States (Davis & Pase, 1977; Hallgreen, 2004). Similar compartmentalization mechanisms could be probably observed in pioneer (*P. cooperi*, *P. duranguensis*) in contrast to secondary pine species of succession (*P. ayacahuite*, *P. teocote*, *P. menziesii*).

Asynchrony in the use of resources would also likely explain how complex forests increase forest productivity (Tilman et al., 1997; Hector et al., 1999). Although there is no information on this issue for the local species in this study, Merlin-Bermúdez & Návar (2005) and Návar (2014) modeled growth of oak species and reported that *Q. sideroxylla* grow at a slower rate but for longer periods of time than pine trees regardless of the similitude of diameter structures. Because of the differential root systems, pines transpire most soil water during the rainy, growing season dependent on the total depth of rainfall; while oaks would probably transpire during most of the growing season but at a smaller rate independent of the total seasonal precipitation.

Asynchrony in the use of resources may also explain part of the tree growth variability and the lack of competition between pioneer (pines) and secondary (oaks) species of succession. Návar et al. (2004); Návar (2014) noted that the diameter distributions of pine and oaks of the Western Sierra Madre Mountain Range grow independently stressing the potential lack of competition for resources between these two groups of tree species. In the Eastern Sierra Madre Mountain Range, oak removal from mixed stands reduced basal area to 50% but residual pines, with an average age of 40 years, did not respond by reducing or accelerating diameter growth (Domínguez & Návar, 1993); neither regenerated openings with pine seedlings (De Los Ríos, 2001). In the Southern Sierra Madre Mountain Range, in old forest stands dominated by oaks, González et al. (1995) and Richardson & Bond (1991) observed selective oak harvesting and overgrazing practices favor the establishment of pioneer pine trees. These practices control oak abundance and diversity and increase tree diversity and forest productivity.

Compartmentalization and the asynchrony in the use of resources among tree species make forest communities to be stable in the long run. In fact, research have supported these findings that tree diversity and canopy structural complexity enhance ecosystem reliability (Naeem & Shibin, 1997) and buffer against perturbations (Naeem et al., 1994; Schulze & Mooney, 1994). Therefore the management of native forests with emphasis on uneven-aged forests at differential spatial scales, rather than on even-aged forests at large tracts of forests, must be carried out in order to conserve tree diversity and structural complexity of native coniferous forests of the Sierra Madre Occidental Mountain Range. The alternate management practices following these rules would also help forest reliability and resilience to modern as well as to conventional forest disturbances. Visual observations and the quantification of tree mortality by heat waves coupled with acute dry episodes revealed the preferment mortality of trees growing in mono-specific stands. Tree die back by these disturbances appears to be lessened in native, mixed, complex forests maybe because oaks transpire water extracted by the long-tap root systems, taping water resources from deep soil profiles, from shallow to deep aquifers or from nearby creeks. This extra-source of water vapor in the air within canopies diminishes the strength of heat waves and reduces atmospheric water demand from pines as well. Then, these silvicultural prescriptions must aim at this time to buffer preferentially against these modern climatic perturbations as they have been ravaging forests elsewhere (Hunter, 1999).

### Conclusions and recommendations

Observations derived from this research reveal forest productivity can be optimized with silvicultural prescriptions that must aim to balance the mixture of pines and oaks, as well as to conserve the structural complexity in the vertical and horizontal dimensions of trees. Tree diversity should follow a well-balanced combination of species richness and abundance that resembles intermediate rather than a pioneer stage of tree succession, mimicking probably diversity-abundance structures predicted by broken stick models. Top height structural complexity should be skewed with high variation (between 2.5 to 30 m), while diameter distributions skewed to the right. These recommendations are supported by the statistical relationships of forest productivity, tree diversity, and canopy structural complexity. These silvicultural prescriptions could preferentially buffer against contemporaneous perturbations associated with coupled heat waves and dry spells.

##  Supplemental Information

10.7717/peerj.7051/supp-1 Supplemental Information 1 Raw data

## Additional Information and Declarations

### Data Availability

The following information was supplied regarding data availability: The raw data is available in a Supplemental File.

## References

[ref-1] Allen CD, Macalady AK, Chenchouni H, Bachelet D, McDowell N, Vennetier M, Kitzberger T, Rigling A, Breshears D, Hoggi EH, Gonzalez P, Fensham R, Zhang Z, Castro J, Demidova N, Lim JH, Allard G, Running SW, Semerci A, Cobb N (2010). A global overview of drought and heat-induced tree mortality reveals emerging climate change risks for forests. Forest Ecology and Management.

[ref-2] Berg DR, Brown TK, Blessing B (1996). Silvicultural systems design with emphasis on the forest canopy. Northwest Science.

[ref-3] Bonan GB (2008). Forests and climate change: forcing, feedbacks, and the climate benefits of forests. Science.

[ref-4] Carey AB, Wilson SM (2001). Induced spatial heterogeneity in forest canopies: responses of small mammals. Journal of Wildlife Management.

[ref-5] Clark DA, Brown S, Kicklighter DW, Tomlison JQ, Ni J (2001). Measuring net primary production in forests; concepts and field methods. Ecological Applications.

[ref-6] Clutter JL, Forston JC, Pienaar LV, Brister GH, Bailey RL (1983). Timber management: a quantitative approach.

[ref-7] Compean-Guzman J (1996). La importancia de la caracterización tecnológica de la madera y su relación con la clasificación estructural. El caso de Pinus cooperii. Ubamari.

[ref-8] Contreras-Aviña JC, Návar-Cháidez J (2002). Ecuaciones aditivas para estimar componentes de volumen para Pinus teocote de Durango, México. Ciencia Forestal en Mexico.

[ref-9] Davalos SR, Wangaard FF, Echenique-Manriquez R (1977). La madera y su uso en la construcción. Clasificación de la madera de pinos mexicanos. INIREB. No 2.

[ref-10] Davis EA, Pase CP (1977). Root system of shrub live oak: implications for water yield in Arizona chaparral. Journal of Soil and Water Conservation.

[ref-11] De Los Ríos CE (2001). Efectos de la intensidad de corta y tratamientos al suelo en la regeneración natural de *Pinus psedostrobus* Lindl. en el ejido Purisima, Iturbide, Nuevo León, México. Tesis Profesional de Ingeniería Forestal.

[ref-12] Domínguez PA, Návar J (1993). El efecto de los aclareos en el crecimiento radial en un bosque de pino-encino en Iturbide, Nuevo León. Resumen de Ponencias.

[ref-13] Franklin JP, Perry DA, Schowalter J, Harmon ME, McKee A, Spies TA, Perry DA, Meurisse R, Thomas B, Miller R, Boyle J, Means J, Perry CR, Powers RF (1989). Importance of ecological diversity in maintaining long-term site productivity. Maintaining the long-term productivity of Pacific Northwest forest ecosystems.

[ref-14] González EM, Ramírez NM, Quintana PF, Martínez M (1995). La utilización de los encinos y la conservación de la biodiversidad en los Altos de Chiapas. Memorias. III Seminario Nacional sobre Utilización de Encinos. Linares, N.L. 4–6 Noviembre de 1992.

[ref-15] Graciano JJ (2001). Técnicas de evaluación dasométrica y ecológica de los bosques de coníferas bajo manejo de la Sierra Madre Occidental del Centro-Sur de Durango, México. Tesis de Maestria en Cienicas.

[ref-16] Haan CT (2003). Statistical methods in hydrology.

[ref-17] Hahn GJ, Shapiro SS (1967). Statistical models in engineering.

[ref-18] Hallgreen S (2004). Tree physiology: shoot growth and canopy development.

[ref-19] Hartley MJ (2002). Rationale and methods for conserving biodiversity in plantation forests. Forest Ecology and Management.

[ref-20] Hector A, Schmid B, Beierkuhnlein H, Caldeira MC, Diemer M, Dimitrakopoulos M, Finn JA, Freitas H, Giller PS, Good J, Harris A, Hogberg P, Huss-Uanell P, Joshi J, Jumpponen M, Korner C, Leadley P, Loreau M, Minns A, Mulder CPH, O’Donovan G, Otway SJ, Pereira SJ, Prinz A, Read DJ, Scherer-Lorenzen M, Schulze D, Siamantziouras M, Spehn M, Terry AM, Troumbis AM, Woodward M, Yachi S, Lawton JH (1999). Plant diversity and productivity experiments in European grasslands. Science.

[ref-21] Hiura T (2001). Stochasticity of species assemblage of canopy trees and understory plants in a temperate secondary forest created by major disturbances. Ecological Research.

[ref-22] Hooper DU, Vitousek PM (1997). The effects of plant composition and diversity on ecosystem processes. Science.

[ref-23] Hunter MLJ (1999). Maintainting biodiversity in forest ecosystems.

[ref-24] Huston MA (1994). Biological diversity; the coexistance of species in changing landscapes.

[ref-25] Ishii H, Ford ED, Dinnie DE (2002). The role of epicormic shoot production in maintaining foliage in old Pseudotsuga menziesii (Douglas-fir) trees II. Basal reiteration from older branch axes. Canadian Journal of Botany.

[ref-26] Ishii H, Reynolds JH, Ford ED, Shaw DC (2000). Height growth and vertical development of an old-growth Pseudotsuga-Tsuga forest in southwestern Washington State, USA. Canadian Journal of Forest Research.

[ref-27] Kelty MJ, Kelty MJ, Larson BC, Oliver CD (1992). Comparative productivity of monocultures and mixed-species stands. P. 125–141 in the ecology and silviculture of mixed species forests.

[ref-28] Leibold MA (1999). Biodiversity and nutrient enrichment in pond plankton communities. Evolutionary Research.

[ref-29] Magurran AE (2004). Diversidad ecológica y su medición.

[ref-30] McArthur RH (1957). On the relative abundance of bird species. Proceedings of the National Academy of Sciences of the United States of America.

[ref-31] Merlin-Bermúdez E, Návar J (2005). Desarrollo de un modelo de rendimiento e incremento para Quercus sideroxylla en bosques mixtos de Durango, México. Agrofaz.

[ref-32] Mittelbach GG, Steiner CF, Scheiner SM, Gross KL, Reynolds HL, Waide RB, Willig MR, Dodson SI (2001). What is the observed relationship between species richness and productivity?. Ecology.

[ref-33] Naeem S, Shibin L (1997). Biodiversity enhances ecosystem reliability. Nature.

[ref-34] Naeem S, Thompson LJ, Lawler SP, Lawton JH, Woodfin RM (1994). Declining biodiversity can alter the performance of ecosystems. Nature.

[ref-35] Návar J (2008). Reconstrucción de las sequías en los últimos 10 mil años en el norte de México. AGROFAZ.

[ref-36] Návar J (2009). Alometric equations for tree species and carbon stocks for forests of northwestern Mexico. Forest Ecology and Management.

[ref-37] Návar J (2014). A stand-class growth and yield model for Mexico’s northern temperate, mixed and multiaged forests. Forests.

[ref-38] Návar J (2015). Hydro-climatic variability and perturbations in Mexico’s northwestern temperate forests. Ecohydrology.

[ref-39] Návar J, Contreras AJC (2000). Ajuste de la distribución Weibull a las estructuras diamétricas de rodales irregulares de Pino de Durango, México. Agrociencia.

[ref-40] Návar J, González N, Maldonado D, Graciano J, Dale V, Parresol B (2004). Additive biomass equations for pine species of forest plantations of Durango, Mexico. Madera y Bosques.

[ref-41] O’Hara K (2016). What is close-to-nature silviculture in a changing world. Forestry.

[ref-42] Parker GG, Lowman MD, Naclcarni NM (1995). Structure and microclimate of forest canopies. P. 730-0 106 in Forest canopies.

[ref-43] Raffa KF, Aukema BH, Bentz BJ, Carroll AL (2008). Cross-scale drivers of natural disturbances prone to anthropogenic amplification: the dynamics of bark beetle eruptions. Bioscience.

[ref-44] Richardson DM, Bond WJ (1991). Determinants of plant distribution: evidence from pine invasions. American Naturalist.

[ref-45] Schulze ED, Mooney HA (1994). Biodiversity and ecosystem function.

[ref-46] Staudhammer CL, Lemay VM (2001). Introduction and evaluation of possible indices of stand structural diversity. Canadian Journal of Forest Research.

[ref-47] Stevens MHH, Carson WP (1999). Plant density determines species richness along an experimental fertility gradient. Ecology.

[ref-48] Stevens MHH, Carson WP (1999a). The significance of assemblage-level thinning for species richness. Journal of Ecology.

[ref-49] Tilman D, Knops J, Wedin D, Reich P, Ritchie M, Seimann E (1997). The influence of functional diversity and composition on ecosystem processes. Science.

[ref-50] Van Mantgem P, Stephenson NL, Byrne JC, Daniles LD, Franklin JF, Fulé P, Harmon ME, Larson AJ, Smith JM, Taylor AH, Veblen TT (2009). Widespread increase of tree mortality rates in the western United States. Science.

[ref-51] Vanclay KV (1994). Modelling forest growth and yield: applications to mixed tropical forests.

[ref-52] Wright SJ (2001). Plant diversity in tropical forests: a review of mechanisms of species coexistence. Oecologia.

